# Combined SIRT3 and SIRT5 deletion is associated with inner retinal dysfunction in a mouse model of type 1 diabetes

**DOI:** 10.1038/s41598-019-40177-6

**Published:** 2019-03-07

**Authors:** Jonathan B. Lin, Joseph B. Lin, Howard C. Chen, Teresa Chen, Rajendra S. Apte

**Affiliations:** 1 Department of Ophthalmology & Visual Sciences, Washington University School of Medicine, St. Louis, MO USA; 2 Neuroscience Graduate Program, Division of Biology and Biomedical Sciences, Washington University School of Medicine, St. Louis, MO USA; 3 Department of Developmental Biology, Washington University School of Medicine, St. Louis, MO USA; 4 Department of Medicine, Washington University School of Medicine, St. Louis, MO USA

## Abstract

Diabetic retinopathy (DR) is a major cause of blindness in working adults in the industrialized world. In addition to vision loss caused by macular edema and pathological angiogenesis, DR patients often exhibit neuronal dysfunction on electrophysiological testing, suggesting that there may be an independent neuronal phase of disease that precedes vascular disease. Given the tremendous metabolic requirements of the retina and photoreceptors in particular, we hypothesized that derangements in metabolic regulation may accelerate retinal dysfunction in diabetes. As such, we induced hyperglycemia with streptozotocin in mice with monoallelic *Nampt* deletion from rod photoreceptors, mice lacking SIRT3, and mice lacking SIRT5 and tested multiple components of retinal function with electroretinography. None of these mice exhibited accelerated retinal dysfunction after induction of hyperglycemia, consistent with normal-appearing retinal morphology in hyperglycemic *Sirt3*^−/−^ or *Sirt5*^−/−^ mice. However, mice lacking both SIRT3 and SIRT5 (*Sirt3*^−/−^*Sirt5*^−/−^ mice) exhibited significant evidence of inner retinal dysfunction after induction of hyperglycemia compared to hyperglycemic littermate controls, although this dysfunction was not accompanied by gross morphological changes in the retina. These results suggest that SIRT3 and SIRT5 may be involved in regulating neuronal dysfunction in DR and provide a foundation for future studies investigating sirtuin-based therapies.

## Introduction

Diabetic retinopathy (DR) is a major cause of blindness in working adults in the industrialized world^1^. Patients with early-stage disease can exhibit non-proliferative DR, which consists of initial pericyte loss and microaneurysms, followed by capillary wall damage and retinal edema or hemorrhages. If it is left untreated, proliferative DR ensues, leading to hypoxic pathologic neovascularization and further vision loss. Current therapeutic strategies for DR target vascular endothelial growth factor (VEGF)^2^. While anti-VEGF therapies have greatly improved treatment outcomes, they are not always effective and are sometimes associated with adverse events^3–7^. Importantly, anti-VEGF therapies do not address the possibility that neuroretinal dysfunction may contribute to vision loss independently from vascular disease. If there is indeed independent neuronal disease, current therapies directed against pathological angiogenesis leave this retinal neuron death completely unabated. Some groups have also suggested that prolonged VEGF blockade in the retina may affect the neuroprotective functions of VEGF that are important in retinal physiology^8,9^, although not all studies are in agreement^10^.

In support of the possibility of an independent neuronal component of disease, there have been numerous reports that DR patients exhibit early deficits on electrophysiological testing, such as delayed implicit times and decreased oscillatory potential amplitudes, observed prior to any evidence of vascular dysfunction^11–15^. Moreover, other studies have found that various rodent models of diabetes, including high-fat diet-induced metabolic dysfunction and streptozotocin (STZ)-induced hyperglycemia, also exhibit electrophysiological changes that are indeed suggestive of retinal neuron dysfunction independent from vascular disease^14,16,17^. Therefore, there is a great need not only to characterize the neuroretinal dysfunction associated with DR but also to identify the underlying pathogenic mechanisms to facilitate the discovery of novel approaches for targeted interventions.

Past studies have shown that, in the STZ-induced model of diabetes, prolonged systemic hyperglycemia leads to retinal oxidative stress and retinal mitochondrial damage, which ultimately cause retinal neuron death^18–20^. Therefore, we hypothesized that impaired mitochondrial function broadly contributes to retinal neurodegeneration in DR. This hypothesis is consistent with the fact that the retina and photoreceptors in particular have high bioenergetic demands not only to maintain a constant dark current but also to meet their anabolic needs due to constant membrane turnover, which limit their capacity to tolerate metabolic perturbations^21,22^.

Since we recently demonstrated that NAMPT-mediated NAD^+^ biosynthesis, SIRT3, and SIRT5 all play important roles in maintaining retinal energetic homeostasis^23^, we hypothesized that these pathways may also play neuroprotective roles in DR. In this study, we investigated whether partially disrupting NAMPT-mediated NAD^+^ biosynthesis or deleting SIRT3/SIRT5 would accelerate early neuroretinal dysfunction in the STZ-induced mouse model of type 1 diabetes. In contrast with our hypothesis, we found that abrogating these pathways individually did not accelerate neuroretinal dysfunction in mice based on any of the electrophysiological parameters that we tested. On the other hand, mice lacking both SIRT3 and SIRT5 exhibited significantly more inner retinal dysfunction following induction of hyperglycemia compared to hyperglycemic littermate controls. Together, these findings suggest that SIRT3 and SIRT5 may have potential redundant neuroprotective roles in a mouse model of type 1 DR.

## Results

### Monoallelic *Nampt* deletion in rod photoreceptors does not accelerate retinal dysfunction in a mouse model of type 1 diabetes

**Figure 1:**
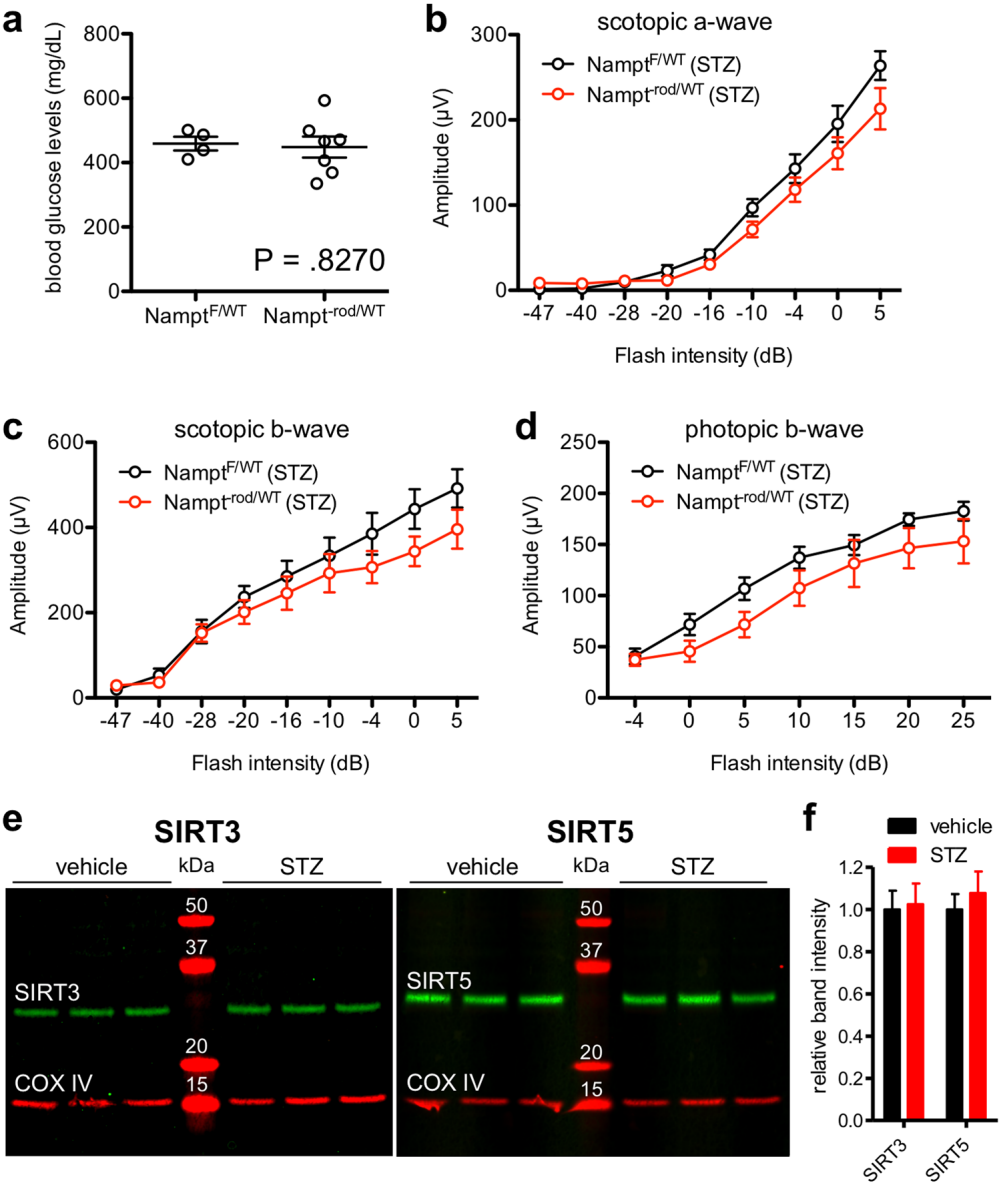
(**a**) Mice with monoallelic *Nampt* deletion from rod photoreceptors (*Nampt*^−rod/WT^; N = 7) were similarly susceptible to streptozotocin (STZ)-induced hyperglycemia compared to *Nampt*^F/WT^ controls (N = 4; 2-tailed, unpaired t-test). (**b**–**d**) At 11 weeks after STZ induction, *Nampt*^−rod/WT^ mice (N = 6–7) did not exhibit any signs of retinal dysfunction compared to *Nampt*^F/WT^ controls (N = 4) based on their scotopic a-wave, scotopic b-wave, or photopic b-wave amplitudes (2-way mixed ANOVA). (**e**,**f**) At 6 weeks after STZ induction in wild-type (WT) mice, there were no changes in retinal SIRT3 or SIRT5 protein levels as measured by immunoblotting shown in **(e**) with quantifications shown in (**f**). Open circles depict individual mice (**a**); graphs depict mean ± S.E.M. (**b**–**d**) or mean + S.E.M. (**f**) Each lane represents an individual mouse; the entire gel is depicted without splicing or cropping (**e**).

Biallelic *Nampt* deletion in rod or cone photoreceptors leads to rapid retinal degeneration that is complete by 6 weeks of age^23^. However, monoallelic, rod-specific *Nampt* deletion does not lead to significant retinal degeneration as late as six months of age^24^, although this does not rule out the possibility that *Nampt* becomes essential under conditions of metabolic stress, such as in diabetes. Therefore, we sought to determine whether monoallelic *Nampt* deletion in rod photoreceptors (*Nampt*^−rod/WT^) would render rod photoreceptors more vulnerable to degeneration in a mouse model of type 1 diabetes. Both *Nampt*^−rod/WT^ mice and their Cre-negative controls (*Nampt*^F/WT^) were equally susceptible to STZ-induced hyperglycemia (Fig. 1a). We tested the retinal function of these mice with electroretinography (ERG) 11 weeks after STZ induction. We found that there were no significant differences in scotopic a-wave, scotopic b-wave, or photopic b-wave amplitudes (Fig. 1b–d). These findings suggest that monoallelic *Nampt* deletion in rod photoreceptors does not make mice more susceptible to retinal neurodegeneration in a model of type 1 diabetic retinopathy. Thus, *Nampt* is haplosufficient in this disease context.

### SIRT3 and SIRT5 individually play minimal neuroprotective roles in a mouse model of type 1 diabetes

The fact that monoallelic *Nampt* deletion did not accelerate retinal neurodegeneration in a mouse model of type 1 diabetes does not completely rule out the possibility that SIRT3 and SIRT5, downstream sensors of NAD^+^ availability, may still play a role in DR. Because past studies have demonstrated that chronic systemic hyperglycemia induces mitochondrial damage^18–20^, we first evaluated whether there is increased SIRT3 or SIRT5 protein expression in retinas from STZ-induced hyperglycemic, wild-type mice. SIRT3 and SIRT5 were not individually upregulated at the protein level in retinas from hyperglycemic mice compared to those from normoglycemic controls (Fig. 1e,f).

**Figure 2:**
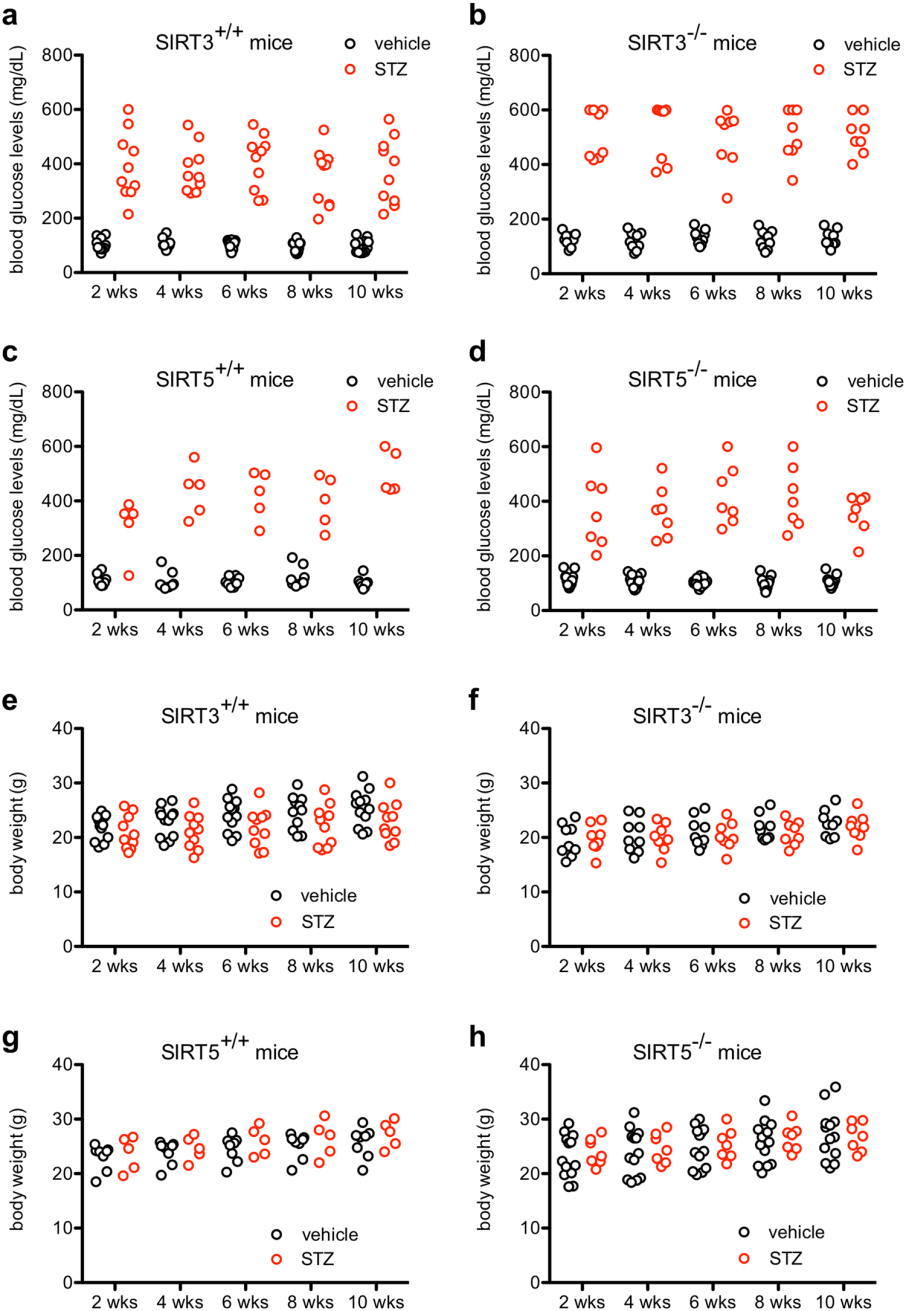
(**a**,**b**) SIRT3 germline knockout (*Sirt3*^−/−^) mice (N = 8–9/group) were similarly susceptible to streptozotocin (STZ)-induced hyperglycemia compared to wild-type (*Sirt3*^+/+^) controls (N = 10–12/group). (**c**,**d**) SIRT5 germline knockout (*Sirt5*^−/−^) mice (N = 7–13/group) were similarly susceptible to STZ-induced hyperglycemia compared to wild-type (*Sirt5*^+/+^) controls (N = 5–8/group). (**e**,**f**) *Sirt3*^+/+^ and *Sirt3*^−/−^ mice (N = 8–12/group) had similar patterns of weight gain after STZ induction. (**g**,**h**) *Sirt5*^+/+^ and *Sirt5*^−/−^ mice (N = 5–13/group) had similar patterns of weight gain after STZ induction. Open circles depict individual mice (**a**–**h**).

**Figure 3:**
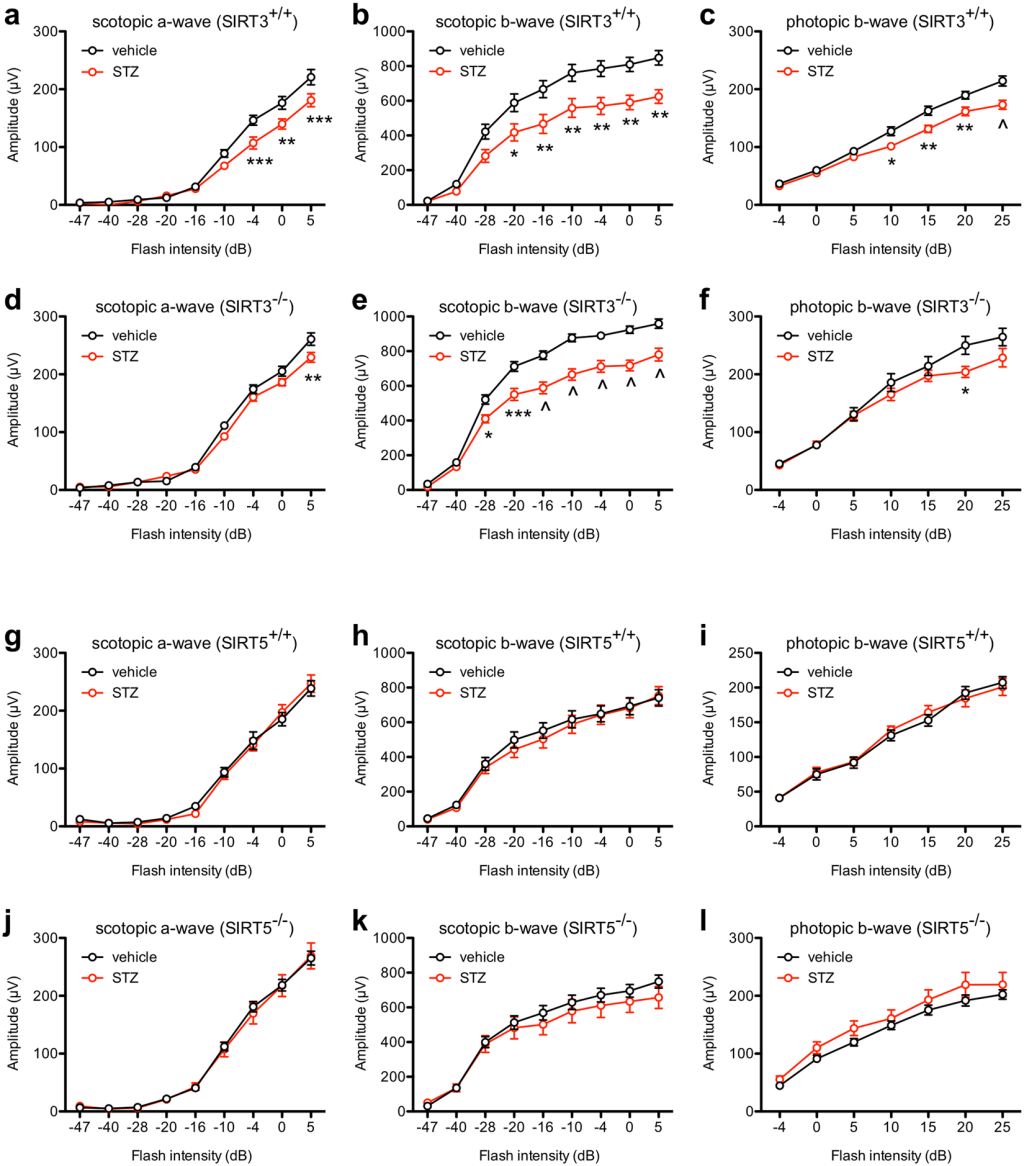
(**a**–**f**) At 11 weeks after streptozotocin (STZ) induction, hyperglycemic SIRT3 germline knockout (*Sirt3*^−/−^) mice had significant retinal dysfunction based on their scotopic a-wave, scotopic b-wave, and photopic b-wave amplitudes compared to normoglycemic mice of the same genotype (N = 8–9/group; 2-way mixed ANOVA with Bonferroni post-hoc test). Hyperglycemic wild-type (*Sirt3*^+/+^) mice also exhibited similar retinal dysfunction compared to normoglycemic controls of the same genotype (N = 11–12/group; 2-way mixed ANOVA with Bonferroni post-hoc test). (**g**–**l**) At 11 weeks after STZ induction, hyperglycemic SIRT5 germline knockout (*Sirt5*^−/−^) and wild-type (*Sirt5*^+/+^) mice did not exhibit any retinal dysfunction compared to normoglycemic controls of the same genotype (N = 8–13/group; 2-way mixed ANOVA). Graphs depict mean ± S.E.M. (**a**–**l**) (*P < 0.05; **P < 0.01; ***P < 0.001; ^P < 0.0001).

To test whether SIRT3 or SIRT5 exert a neuroprotective role under hyperglycemic conditions, we characterized whether germline deletion of *Sirt3* or *Sirt5* caused early neuroretinal dysfunction in a mouse model of type 1 diabetes. We found that mice lacking *Sirt3* (*Sirt3*^−/−^) or *Sirt5* (*Sirt5*^−/−^) were equally susceptible to STZ-induced hyperglycemia compared to wild-type controls (Fig. 2a–d). Moreover, all mice, regardless of genotype or glycemic status, had similar patterns of weight gain over time (Fig. 2e–h), suggesting a similar systemic burden of disease. When we tested retinal function with ERG, we found that hyperglycemic *Sirt3*^+/+^ and *Sirt3*^−/−^ mice exhibited modest retinal dysfunction compared to their normoglycemic controls in their scotopic a-wave, scotopic b-wave, and photopic b-wave amplitudes (Fig. 3a–f). However, of interest, the magnitude of this difference between STZ- and vehicle-treated mice was similar for both genotypes, suggesting a minimal neuroprotective role for SIRT3. In contrast, hyperglycemic *Sirt5*^+/+^ and *Sirt5*^−/−^ mice did not exhibit retinal dysfunction compared to their normoglycemic controls in their scotopic a-wave, scotopic b-wave, or photopic b-wave amplitudes (Fig. 3g–l).

**Figure 4:**
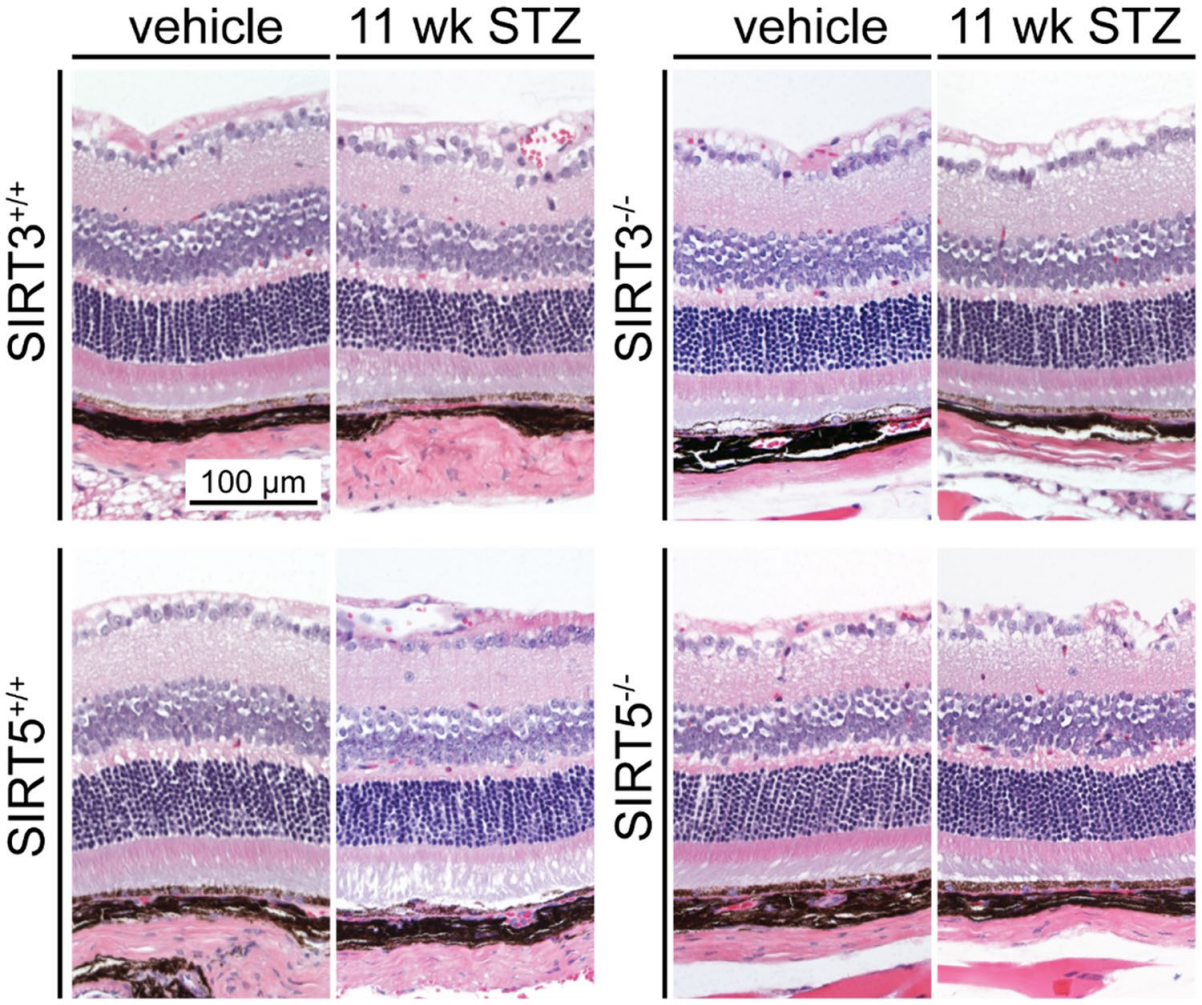
Representative histological images demonstrate that there were no changes in retinal morphology upon germline deletion of either SIRT3 or SIRT5, regardless of whether the mice were normo- or hyperglycemic.

In agreement with this electrophysiological characterization showing similar and only moderate hyperglycemic-induced changes in retinal function between *Sirt3*^+/+^ and *Sirt3*^−/−^ mice, we found no gross differences in retinal morphology between these mice upon histological examination (Fig. 4). Similarly, there were no gross morphological changes between *Sirt5*^+/+^ and *Sirt5*^−/−^ retinas, regardless of whether they were from normo- or hyperglycemic mice (Fig. 4). These data suggest that SIRT3 and SIRT5 individually play only minimal, if any, neuroprotective roles under conditions of hyperglycemia.

**Figure 5:**
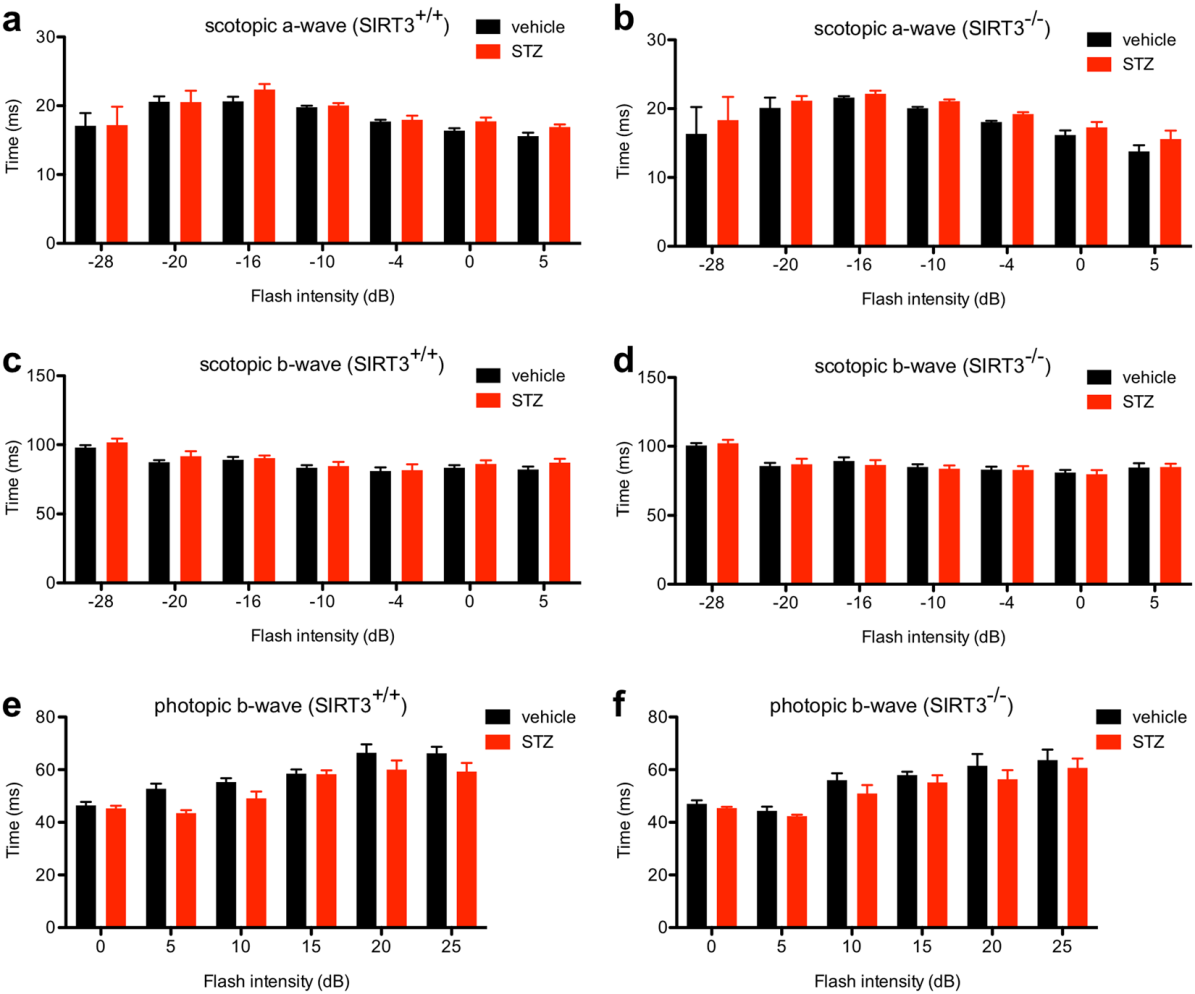
(**a**–**d**) There were no significant differences in the scotopic a- or b-wave implicit times when comparing hyperglycemic SIRT3 germline knockout (*Sirt3*^−/−^) and wild-type control (*Sirt3*^+/+^) mice to normoglycemic mice of the same genotype (N = 8–12/group; 2-way mixed ANOVA). (**e**,**f**) Hyperglycemic *Sirt3*^+/+^ mice had mildly reduced photopic b-wave implicit times compared to normoglycemic *Sirt3*^+/+^ mice. Although not statistically significant, there was a similar effect size in hyperglycemic versus normoglycemic *Sirt3*^−/−^ mice (N = 8–12/group; 2-way mixed ANOVA). Graphs depict mean + S.E.M. (**a**–**f**).

**Figure 6:**
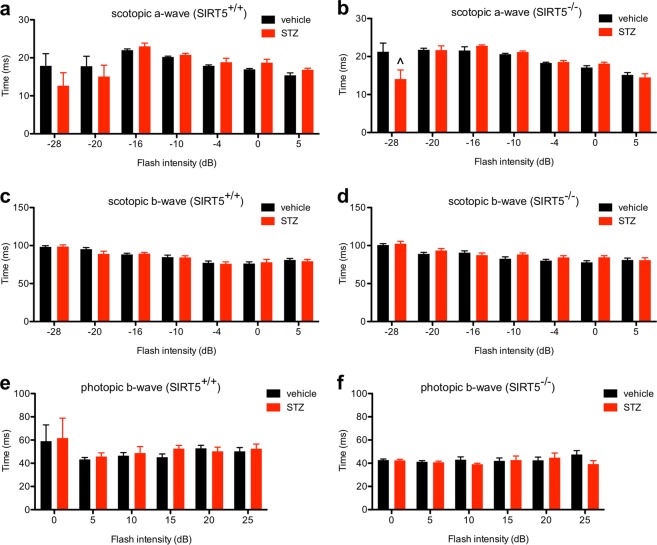
(**a**–**f**) There were no significant differences in the scotopic a-wave, scotopic b-wave, or photopic b-wave implicit times when comparing hyperglycemic SIRT5 germline knockout (*Sirt5*^−/−^) and wild-type control (*Sirt5*^+/+^) mice to normoglycemic mice of the same genotype except for one pairwise comparison at a single flash intensity after post-hoc testing (N = 8–13/group; 2-way mixed ANOVA with Bonferroni post-hoc test). Graphs depict mean + S.E.M. (**a**–**f**) (^P < 0.0001).

Since past reports have suggested that mouse models of DR may exhibit subtle changes in ERG responses, we also assessed whether there were any changes in a- or b-wave implicit times. We did not find any significant differences in scotopic a- or b-wave implicit times in hyperglycemic versus normoglycemic *Sirt3*^+/+^ and *Sirt3*^−/−^ mice (Fig. 5a–d). Photopic b-wave implicit times were mildly reduced when comparing hyperglycemic *Sirt3*^+/+^ mice to normoglycemic *Sirt3*^+/+^ mice (main effect_STZ_: P = 0.0135; Fig. 5e). Although not statistically significant, hyperglycemic versus normoglycemic *Sirt3*^−/−^ mice showed a similar magnitude of change in photopic b-wave implicit times, arguing for a minimal neuroprotective role for SIRT3 (Fig. 5f). Likewise, except for one pairwise comparison after post-hoc testing, there were no significant differences in scotopic a-wave, scotopic b-wave, or photopic b-wave implicit times in hyperglycemic *Sirt5*^+/+^ or *Sirt5*^−/−^ mice compared to normoglycemic mice of the same genotype (Fig. 6a–f). These data suggest, once again, that SIRT5 plays a minimal neuroprotective role.

**Figure 7:**
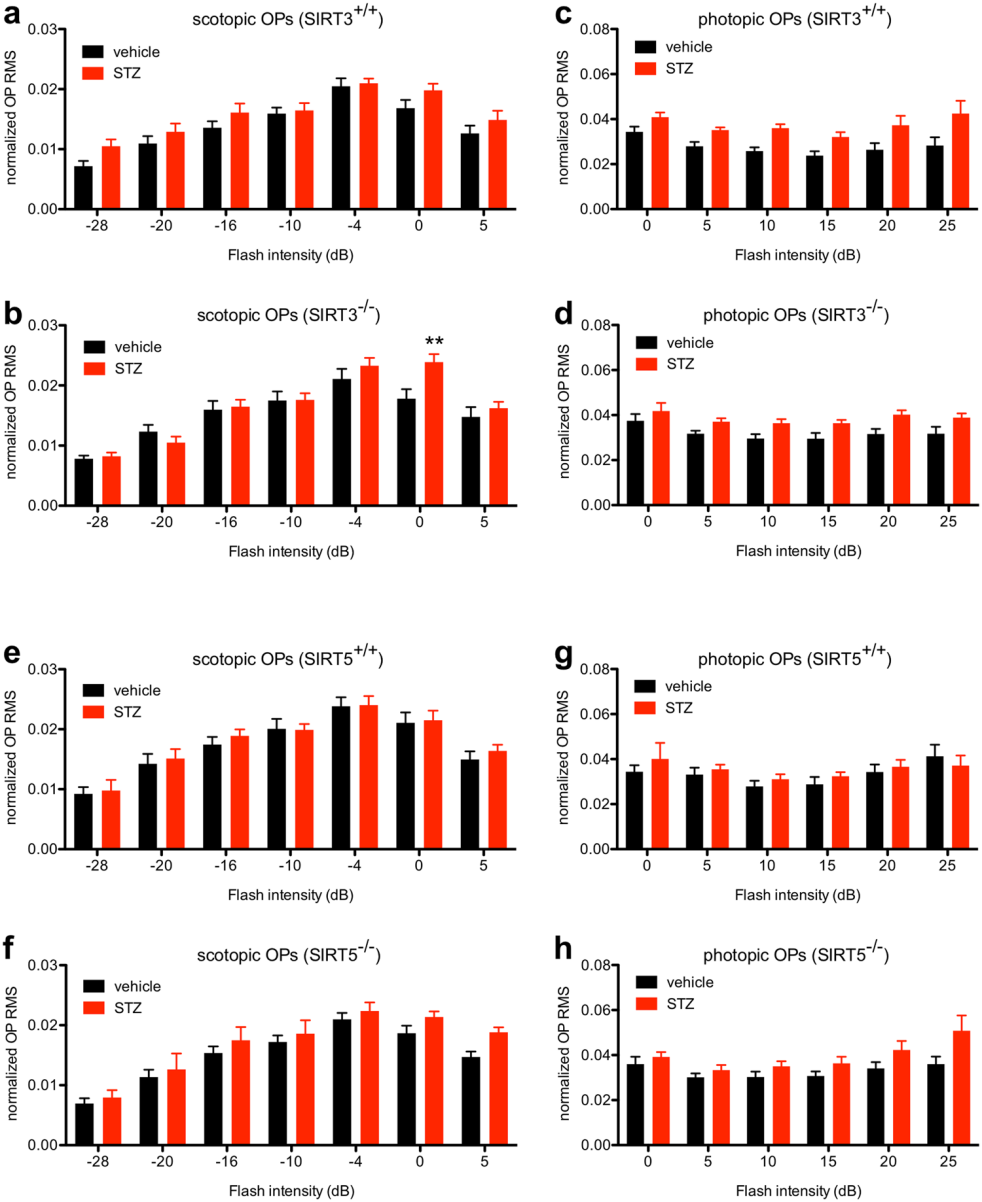
(**a**,**b**) There were no significant differences in scotopic oscillatory potential (OP) amplitudes between hyperglycemic SIRT3 germline knockout (*Sirt3*^−/−^) and wild-type control (*Sirt3*^+/+^) mice and normoglycemic mice of the same genotype except for one pairwise comparison at a single flash intensity after post-hoc testing (N = 8–12/group; 2-way mixed ANOVA with Bonferroni post-hoc test). (**c**,**d**) Although photopic OP amplitudes were significantly increased in hyperglycemic versus normoglycemic *Sirt3*^−/−^ mice, we observed a similar pattern in hyperglycemic versus normoglycemic *Sirt3*^+/+^ mice (N = 8–12/group; 2-way mixed ANOVA). (**e**–**h**) There were no significant differences in scotopic or photopic OP amplitudes in hyperglycemic SIRT5 germline knockout (*Sirt5*^−/−^) and wild-type control (*Sirt5*^+/+^) mice compared to normoglycemic mice of the same genotype (N = 8–13/group; 2-way mixed ANOVA). Graphs depict mean + S.E.M. (**a**–**h**) (RMS: root mean square; **P < 0.01).

We next evaluated oscillatory potential (OP) amplitudes, which may reveal inner retinal dysfunction. There were no significant differences of appreciable effect size in scotopic OP amplitudes between hyperglycemic *Sirt3*^+/+^ and *Sirt3*^−/−^ mice and normoglycemic mice of the same genotype, except for one pairwise comparison at a single flash intensity after post-hoc testing (Fig. 7a,b). Although photopic OP amplitudes were significantly increased in hyperglycemic versus normoglycemic *Sirt3*^−/−^ mice (main effect_STZ_: P = 0.0113), we observed a similar pattern in hyperglycemic versus normoglycemic *Sirt3*^+/+^ mice (main effect_STZ_: P = 0.0051) (Fig. 7c,d). Finally, there were no differences in scotopic or photopic OPs in hyperglycemic *Sirt5*^+/+^ and *Sirt5*^−/−^ mice compared to normoglycemic mice of the same genotype (Fig. 7e–h). Together, these findings further support our assertion that SIRT3 and SIRT5 individually play minimal neuroprotective roles in a mouse model of DR.

### Combined SIRT3 and SIRT5 deletion is associated with inner retinal dysfunction in a mouse model of type 1 diabetes

**Figure 8:**
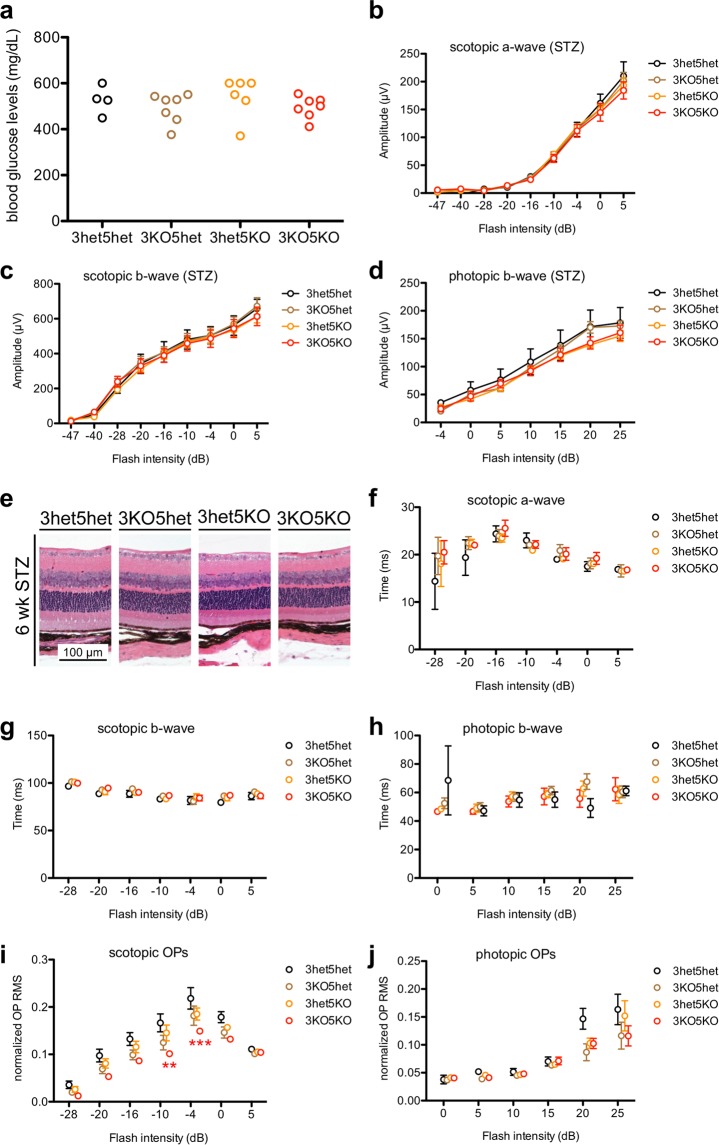
(**a**) SIRT3/SIRT5 double knockout mice (3KO5KO) were equally susceptible to streptozotocin (STZ)-induced hyperglycemia compared to littermate controls of various genotypes. (**b**–**d**) At 6 weeks after STZ induction, hyperglycemic 3KO5KO mice did not exhibit any retinal dysfunction based on their scotopic a-wave, scotopic b-wave, and photopic b-wave amplitudes compared to hyperglycemic littermate controls (N = 4–7/group; 2-way mixed ANOVA). (**e**) Representative histological images show that hyperglycemic 3KO5KO mice did not exhibit any changes in retinal morphology compared to hyperglycemic littermate controls. (**f**–**h**) Hyperglycemic 3KO5KO mice did not have any significant changes in scotopic a-wave, scotopic b-wave, or photopic b-wave implicit times compared to hyperglycemic littermate controls (N = 4–7/group; 2-way mixed ANOVA). (**i**,**j**) Hyperglycemic 3KO5KO mice had significant reductions in scotopic but not photopic oscillatory potential (OP) amplitudes compared to hyperglycemic littermate controls (N = 4–7/group; 2-way mixed ANOVA with Bonferroni post-hoc test). Open circles depict individual mice (**a**); graphs depict mean ± S.E.M. (**b**–**d**, **f**–**j**) (RMS: root mean square; **P < 0.01; ***P < 0.001; red asterisks indicate significant differences compared to *Sirt3*^+/−^*Sirt5*^+/−^ mice after post-hoc testing).

Numerous past studies have suggested that SIRT3 and SIRT5 may have some degree of redundancy since they are both mitochondrial deacylases with many of the same protein targets^25,26^. Moreover, we have previously reported that although mice lacking both SIRT3 and SIRT5 do not exhibit retinal degeneration at baseline, they are more vulnerable to light-induced degeneration^23^. Therefore, we sought to determine whether combined deletion of both SIRT3 and SIRT5 may render mice more susceptible to retinal dysfunction in a model of type 1 diabetes. Mice lacking both SIRT3 and SIRT5 (*Sirt3*^−/−^*Sirt5*^−/−^) were equally susceptible to STZ-induced hyperglycemia compared to control littermates of various genotypes, including *Sirt3*^+/−^*Sirt5*^+/−^, *Sirt3*^−/−^*Sirt5*^+/−^, and *Sirt3*^+/−^*Sirt5*^−/−^ mice (Fig. 8a). When tested with ERG, hyperglycemic *Sirt3*^−/−^*Sirt5*^−/−^ mice did not exhibit any retinal dysfunction in scotopic a-wave, scotopic b-wave, or photopic b-wave amplitudes at 6 weeks following STZ induction compared to hyperglycemic littermate controls (Fig. 8b–d), consistent with absence of any gross changes in retinal morphology upon histological examination (Fig. 8e). Moreover, hyperglycemic *Sirt3*^−/−^*Sirt5*^−/−^ mice did not show significant changes in a- or b-wave implicit times (Fig. 8f–h). However, hyperglycemic *Sirt3*^−/−^*Sirt5*^−/−^ mice did exhibit reduced scotopic OP amplitudes compared to hyperglycemic *Sirt3*^+/−^*Sirt5*^+/−^ littermate controls, indicative of inner retinal dysfunction, although photopic OP amplitudes were unchanged (Fig. 8i,j). These results suggest that SIRT3 and SIRT5 may play redundant, neuroprotective roles in the inner retina under hyperglycemic conditions.

## Discussion

DR is a major cause of blindness in industrialized nations. Although therapies for DR largely focus on antagonizing VEGF to treat the vascular component of disease, there is increasing evidence that there is independent neuronal disease. Therefore, the motivation behind this study was to test whether there was a neuroprotective role for metabolic regulation via NAMPT-mediated NAD^+^ biosynthesis, SIRT3, or SIRT5 in a mouse model of type 1 diabetes. We have previously identified that NAMPT-mediated NAD^+^ biosynthesis is important for retinal energy homeostasis and that both SIRT3 and SIRT5 are important for protecting the retina against light-induced degeneration^23,27^. In the current study, we found that although these pathways have important roles in regulating some facets of retinal homeostasis, they individually play only minimal neuroprotective roles in retinopathy in a mouse model of type 1 diabetes. Moreover, SIRT3 and SIRT5 may have redundant roles in the inner retina and therefore possess the ability to functionally compensate for one another, as deletion of both was associated with modest, but significant, inner retinal dysfunction in the STZ-induced model of type 1 diabetes.

Although our findings are contrary to our original hypothesis, this study sheds important light on the pathophysiology underlying retinal neuron dysfunction in DR and the role of metabolic regulation in this disease process. NAMPT-mediated NAD^+^ biosynthesis has been reported to be essential in numerous cell types, including but not limited to rod and cone photoreceptors^23^, hippocampal and cortical excitatory neurons^28^, adipocytes^29^ and skeletal muscle^30^. Many of these studies have examined animal phenotypes after cell-specific, biallelic *Nampt* deletion using the Cre-lox system. Although these findings provide important insights that provide a foundation for future research, they also may not be a perfect model for what may be occurring in human disease when NAD^+^ homeostasis is perturbed, as they involve complete abrogation of an important cellular pathway. As such, we tested whether monoallelic *Nampt* deletion from rod photoreceptors was sufficient to render rod photoreceptors more vulnerable to dysfunction in a mouse model of diabetes. Our null results indicate that *Nampt* is haplosufficient in this disease context. These findings suggest that there is likely mitochondrial reserve that enables rod photoreceptors to maintain relative metabolic homeostasis even when challenged by partial disruptions in NAMPT-mediated NAD^+^ biosynthesis.

Furthermore, we found that germline deletion of neither SIRT3 nor SIRT5 individually affected the progression of retinal dysfunction in the STZ-induced model of type 1 diabetes, while combined germline deletion of both SIRT3 and SIRT5 was associated with inner retinal dysfunction under hyperglycemic conditions. In contrast, single SIRT3 germline knockout mice themselves are more vulnerable to light-induced degeneration compared to wild-type controls^27^ and SIRT3/SIRT5 double knockout mice are even more vulnerable compared to either single knockout^23^. These contrasting findings confirm that although there are some similarities in the underlying pathophysiology modeled by light-induced degeneration and STZ-induced hyperglycemia, there are also differences in the cell stress and death pathways that are activated by photopic stimuli versus hyperglycemic stress. Although further studies are necessary to confirm our findings and to provide additional mechanistic insight, our study provides evidence that SIRT3 and SIRT5 may have redundant neuroprotective roles in the inner retina. These findings provide a foundation for future investigation aimed at developing sirtuin-based neuroprotection strategies for DR and other retinal neurodegenerative diseases.

## Methods

### Animals

All animal experiments were approved by the Institutional Animal Care and Use Committee (IACUC) of Washington University in St. Louis and were performed in accordance with all relevant guidelines and regulations. We obtained *Nampt*^flox/flox^ mice from Dr. Shin-ichiro Imai^31^ and Rhodopsin-iCre75 transgenic mice from Dr. Ching-Kang Jason Chen^32^. We obtained *Sirt3*^−/−^ and *Sirt5*^−/−^ mice from Jackson Laboratories (Bar Harbor, ME) and bred them with the appropriate strain-matched, wild-type mice from Jackson Laboratories (129S1/SvImJ for *Sirt3*^−/−^ and B6129SF2/J for *Sirt5*^−/−^). To induce systemic hyperglycemia, we injected mice daily for five days with 65 mg/kg body weight streptozotocin (STZ; Sigma, St. Louis, MO) freshly prepared in citrate buffer (0.10 M; pH 4.5) and compared hyperglycemic mice to vehicle-treated mice receiving citrate buffer alone. We measured blood glucose levels with the GLUCOCARD Vital (Arkray, Edina, MN) and weighed mice regularly, humanely euthanizing any mice exhibiting signs of diabetic ketoacidosis or other distress.

### Electroretinography

We performed electroretinography (ERG) with the UTAS-E3000 Visual Electrodiagnostic System running EM for Windows (LKC Technologies, Gaithersburg, MD), as described previously^23^. We extracted quantitative measurements, such as a- and b-wave amplitudes and implicit times, from the ERG waveforms using a Microsoft Excel macro that defines the peak of the a-wave as the most negative point of the average trace and the peak of the b-wave as the most positive point without subtracting oscillatory potentials. To isolate oscillatory potentials, we used a custom script in MATLAB (Mathworks, Natick, MA) to digitally process the ERG waveforms with a 25 Hz high-pass filter and calculated the root-mean-square (RMS) of the oscillatory potentials after normalizing to b-wave amplitudes.

### Immunoblotting

We compared SIRT3 and SIRT5 protein levels in STZ-induced hyperglycemic versus normoglycemic, wild-type control mice by Western blot. We loaded retinal lysate containing 15 μg protein into individual lanes, separated proteins using SDS-polyacrylamide gel electrophoresis, and then transferred them to a nitrocellulose membrane (0.2 μm, BIO-RAD, Hercules, CA). We then blocked membranes with 2.5% milk in 1X PBS. Next, we incubated membranes at 4 °C overnight with a cocktail of primary antibodies containing 0.1% Tween-20 (v/v); a mouse monoclonal antibody against COX IV (Cell Signaling Technology #11967S, dilution 1:1,000); and either a rabbit monoclonal antibody against SIRT3 (Cell Signaling Technology #5490S, dilution 1:1,000) or a rabbit monoclonal antibody against SIRT5 (Cell Signaling Technology #8782, dilution 1:500). We then incubated membranes with a secondary antibody cocktail containing 0.1% Tween-20 (v/v); goat anti-mouse secondary antibody (IRDye 680CW, LI-COR, dilution 1:5,000); and goat anti-rabbit secondary antibody (IRDye 800RD, LI-COR, dilution: 1:5,000). We visualized blots using an Odyssey CLx Imaging System and quantified protein bands of interest using Image Studio 4.0, normalizing to COX IV band intensity as a loading control. Protein expression of SIRT3 and SIRT5 in hyperglycemic, wild-type mice is provided as a ratio relative to expression in normoglycemic, wild-type mice.

### Histology

After euthanizing the mice, we enucleated the eyes and fixed them in 4% glutaraldehyde for 2 hours and then in 4% paraformaldehyde for 24 hours. Next, we embedded the eyes in methacrylate and prepared six sections of 6–8 μm thickness cut through the optic nerve. We stained slides with H&E and acquired bright-field images with an Olympus BX51 microscope.

### Statistics

We performed statistical analysis with Prism 5 (GraphPad, San Diego, CA), using the appropriate test for each data set. We considered P < 0.05 to be statistically significant. All data generated or analyzed during this study are included in this published article.
